# A mouse model of Timothy syndrome exhibits altered social competitive dominance and inhibitory neuron development

**DOI:** 10.1002/2211-5463.12924

**Published:** 2020-07-19

**Authors:** Shin‐ichiro Horigane, Yukihiro Ozawa, Jun Zhang, Hiroe Todoroki, Pan Miao, Asahi Haijima, Yuchio Yanagawa, Shuhei Ueda, Shigeo Nakamura, Masaki Kakeyama, Sayaka Takemoto‐Kimura

**Affiliations:** ^1^ Department of Neuroscience I Research Institute of Environmental Medicine Nagoya University Nagoya Japan; ^2^ Molecular/Cellular Neuroscience Nagoya University Graduate School of Medicine Nagoya Japan; ^3^ Department of Pathology and Laboratory Medicine Nagoya University Hospital Nagoya Japan; ^4^ Laboratory for Systems Neurosciences and Preventive Medicine Faculty of Human Sciences Waseda University Tokorozawa Japan; ^5^ Research Institute for Environmental Medical Sciences Waseda University Tokorozawa Japan; ^6^ Department of Genetic and Behavioral Neuroscience Gunma University Graduate School of Medicine Maebashi Japan; ^7^ Precursory Research for Embryonic Science and Technology (PRESTO) Japan Science and Technology Agency Saitama Japan

**Keywords:** autism spectrum disorder, IntelliCage, L‐type Ca^2+^ channels, neural circuit formation, social competitive dominance, Timothy syndrome

## Abstract

Multiple genetic factors related to autism spectrum disorder (ASD) have been identified, but the biological mechanisms remain obscure. Timothy syndrome (TS), associated with syndromic ASD, is caused by a gain‐of‐function mutation, G406R, in the pore‐forming subunit of L‐type Ca^2+^ channels, Ca_v_1.2. In this study, a mouse model of TS, TS2‐neo, was used to enhance behavioral phenotyping and to identify developmental anomalies in inhibitory neurons. Using the IntelliCage, which enables sequential behavioral tasks without human handling and mouse isolation stress, high social competitive dominance was observed in TS2‐neo mice. Furthermore, histological analysis demonstrated inhibitory neuronal abnormalities in the neocortex, including an excess of smaller‐sized inhibitory presynaptic terminals in the somatosensory cortex of young adolescent mice and higher numbers of migrating inhibitory neurons from the medial ganglionic eminence during embryonic development. In contrast, no obvious changes in excitatory synaptic terminals were found. These novel neural abnormalities in inhibitory neurons of TS2‐neo mice may result in a disturbed excitatory/inhibitory (E/I) balance, a key feature underlying ASD.

## Graphical abstract

Timothy syndrome (TS) associated with autism spectrum disorder (ASD) is caused by G406R mutation in L‐type Ca^2+^ channels. A TS mouse model, TS2‐neo, showed excess migrating inhibitory neurons, higher number of smaller‐sized inhibitory presynaptic terminals, and high social competitive dominance in the IntelliCage. These findings provide new insights into the Ca^2+^‐dependent developmental mechanisms of inhibitory neurons and the pathophysiology of TS.

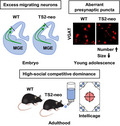

**Abbreviations**
ASD: autism spectrum disorder
GABA: gamma‐aminobutyric acid
NA: numerical aperture
RFID: radiofrequency identification
RT: room temperature
TS: Timothy syndrome
VGAT: vesicular GABA transporter
WT: wild‐type

Autism spectrum disorder (ASD) is a neurodevelopmental disorder marked by behavioral deficits, including impairment of social domain and repetitive/restricted behavior domain. Timothy syndrome (TS) is a multisystem channelopathy that co‐occurs with QT prolongation, arrhythmia, and syndactyly and is highly associated with ASD [1, 2]. TS type 1 (TS1) and TS type 2 (TS2) result from *de novo* missense mutations in *CACNA1C* in either exon 8A [1] or exon 8 [2]. These mutations lead to G406R point mutation in the pore‐forming subunit of the L‐type Ca^2+^ channel, Ca_v_1.2. G406R causes excessive Ca^2+^ currents via deficits of voltage‐ and calcium‐dependent inactivation [1, 3]. A genetically modified knock‐in mouse with heterogeneous TS2 (G406R) point mutation in the L‐type Ca^2+^ channel, TS2‐neo, showed autistic traits of impaired social interaction along with restricted and repetitive/preservative behavior [4, 5].

Ca^2+^ signaling plays a central role in neural circuit formation [6, 7, 8]. Spontaneous Ca^2+^ transients are observed in immature neurons despite the absence of chemical synapses and synaptic inputs. Excitatory and inhibitory neurons that are born in distant areas migrate into the neocortex by two different pathways: radial migration and tangential migration [9]. Importantly, Ca^2+^ influx through L‐type Ca^2+^ channels evoked by excitatory action of ambient gamma‐aminobutyric acid (GABA) and glutamate promotes tangential migration of immature inhibitory neurons, which are born in the medial ganglionic eminence and migrate tangentially into the neocortex [10]. After neuronal migration, Ca^2+^ signaling also contributes to synapse formation and elimination in both excitatory and inhibitory neurons [11, 12]. These processes are believed to be involved in altered spine density and excitatory/inhibitory (E/I) imbalance that are key features underlying ASD [13, 14].

Investigations using mouse models have revealed that the expression of the G406R gain‐of‐function mutation alters several aspects of neural circuit formation of excitatory neurons, including neuronal differentiation, neuronal migration, and dendrite extension [15, 16, 17, 18]. As for inhibitory neurons, studies are limited. A recent study on human iPSC‐derived spheroid showed altered migration in inhibitory neurons [19]. Further studies using animal models are needed to understand how and when the G406R mutation affects the development of inhibitory neurons *in vivo*.

In this study, we aimed to reassess the reported behavioral profiles of TS2‐neo mutant mice using the IntelliCage test system that enables automated behavioral phenotyping in a group housing environment [20, 21] and to visualize potential anomalies in inhibitory neuronal development. In the IntelliCage, TS2‐neo mutant mice showed high social competitive dominance, with preserved behavioral flexibility. After demonstrating behavioral phenotypes, histological analyses of inhibitory neuronal development were performed. We found, in TS2‐neo mice, inhibitory presynaptic puncta were increased in layer (L) 4 but not L2 in the somatosensory cortex on postnatal day (P) 21. This period corresponds to the end of the critical period of developmental maturation of E/I balance [22]. Furthermore, increased numbers of migrating inhibitory neurons were observed in the embryonic brain. Together, these findings present new insights into neural circuit abnormalities especially in inhibitory neurons, which may underlie autistic traits of TS2‐neo mice.

## Materials and methods

### Animals

TS2‐neo mice (B6.Cg‐*Cacna1c^tm2Itl^*/J, Jax stock #019547) [4] were obtained from the Jackson Laboratory (Sacramento, CA, USA), and genotyping PCR was performed following the Jackson Laboratory genotyping protocol. The VGAT‐Venus transgenic mouse line (B6‐Tg(Slc32a1‐YFP*)39Yyan), which expresses Venus [23] under vesicular GABA transporter (VGAT) promoter [24], was used to label inhibitory neurons (hereinafter referred to as VGAT^Venus^ mouse). For VGAT^Venus^ mouse genotyping, Venus fluorescence was checked with LED light and an emission filter (Handy Blue Pro Plus, RelyOn). To analyze tangential migration, heterozygous TS2‐neo mice and the heterozygous VGAT^Venus^ mice were crossed, and embryonic mice were assessed at embryonic day (E) 13.5. Mice for behavior testing were generated via *in vitro* fertilization using C57BL/6J oocytes to obtain offspring. C57BL/6J mice were purchased from Charles River Japan (Yokohama, Kanagawa, Japan). Mice were group‐housed (up to four animals/cage) under 12:12‐h dark–light cycle and had water and food *ad libitum* unless otherwise noted. All animal experiments were conducted following guidelines for care and use of experimental animals of Nagoya and Waseda Universities and were approved by the institutional review committees.

### Behavioral phenotyping by the IntelliCage apparatus

**Fig. 1:**
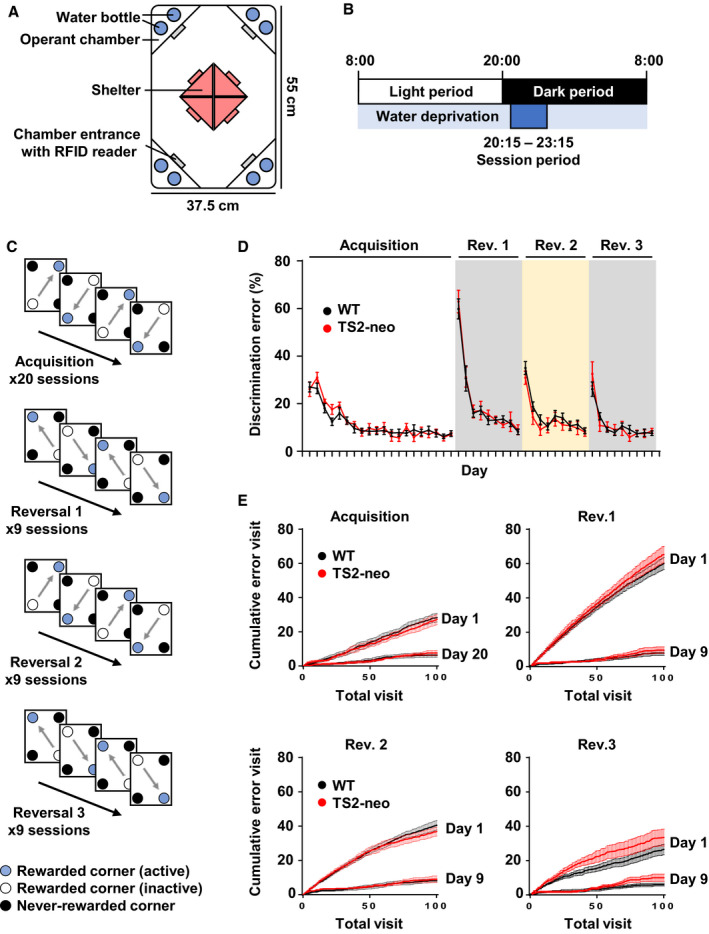
Behavioral phenotyping of TS2‐neo mice in the IntelliCage. (A) Schematic illustration of the IntelliCage apparatus. (B) Daily timeline. (C) Schematic illustrations of a behavioral sequencing task composed of acquisition and reversal blocks to assess flexibility. Acquisition and each reversal block include 20 sessions and 9 sessions, respectively. (D) Learning performance and behavioral flexibility were not affected in TS2‐neo mice. The discrimination error rate was based on the total number of visits to the never‐rewarded corner in the first 100 visits in each session. (E) Cumulative error visits in the first 100 visits in the first and last session of acquisition and reversal blocks. *n* = 8 mice in WT and *n* = 5 in TS2‐neo mice. Data are mean ± SEM.

At an age of P63, mice were anesthetized with isoflurane and a glass‐covered transponder having a unique ID code was implanted subcutaneously for radiofrequency identification (RFID) (Datamars, Temple, TX, USA). Male mice were tested for behavioral flexibility and social competitive dominance behavior in the fully automated IntelliCage (TSE Systems GmbH, Bad Homburg, Germany) (Fig. 1A). The apparatus consists of a polycarbonate cage (55 cm × 37.5 cm × 20.5 cm) containing a triangular operant chamber (15 cm × 15 cm × 21 cm) in each corner, accessible by one mouse at a time. Each chamber allows access to two water bottles for drinking, through a short narrow tunnel equipped with an antenna that reads RFID signals. The behavioral test was conducted during a 3‐h period (20:15–23:15) each day, and mice can access drinking water as a reward only when they showed correct response (a nose‐poking at the assigned corner chamber as the rewarded corner) during test period.

TS2‐neo (*n* = 8) and wild‐type (WT) (*n* = 8) mice were introduced into the apparatus at P126. Mice had free access to food, and the body weights were not different between groups throughout the experimental period (data not shown). Three TS2‐neo mice were excluded because of low locomotor activity with a not evaluable corner visit number. Therefore, TS‐neo (*n* = 5) and WT (*n* = 8) mice were analyzed for behavioral flexibility and social dominance.

The behavioral flexibility test comprised an acquisition phase of place learning and serial reversal learning phases as described previously [20, 21]. In brief, before behavioral flexibility test, mice were allowed to acclimate to the IntelliCage for 8 days with water available *ad libitum* in all corners and then habituated to water restriction for 6 days.

In the acquisition phase, mice were allowed 4‐s access to water as a reward when they visited the assigned corners during the daily 3‐h session period (20:15–23:15) (Fig. 1B). In each session, mice gained rewards continuously by alternating visits between two diagonally positioned rewarded corners (Fig. 1C). Thus, mice had to learn two distantly positioned rewarded corners and shuttle between them. Reward corner assignments were counterbalanced among genotype groups. The acquisition phase included 20 learning sessions. Subsequently, in serial reversal learning, diagonal spatial patterns of the rewarded corners were repetitively reversed every nine sessions (Fig. 1C). Thus, mice had to learn to switch their shuttling behavior between two diagonal spatial patterns. In total, three reversals were conducted in this phase.

Visiting the never‐rewarded corners was regarded as a discrimination error. Numbers of discrimination errors within the first 100 visits in each session were utilized for intersession comparisons of learning performance. Also, to evaluate social competitive dominance for limited water rewards, the duration of each corner visit during the first 10 min in each session during the acquisition phase was used as indices.

### Immunohistochemistry

For presynaptic puncta analysis, mouse brains were collected at P21. After brain fixation and coronal sectioning, brain sections were blocked with 1% bovine serum albumin, 5% normal donkey serum, and 0.3% Triton X‐100 for 1 h at room temperature (RT). Sections were then incubated overnight with mouse monoclonal anti‐VGAT (1 : 500; Synaptic Systems, Göttingen, Germany), goat polyclonal anti‐VGluT1 (1 : 500; Frontier Institute, Ishikari, Hokkaido, Japan), and rabbit polyclonal anti‐VGluT2 (1 : 500; Frontier Institute) diluted in the blocking solution at 4 °C. After washing with PBS, tissue sections were incubated with secondary antibodies, an Alexa Fluor 555‐conjugated donkey anti‐mouse antibody (1 : 500; Molecular Probes, Eugene, OR, USA), an Alexa Fluor 488‐conjugated donkey anti‐goat antibody (1 : 500; Molecular Probes), and an Alexa Fluor 647‐conjugated donkey anti‐rabbit antibody (1 : 500; Molecular Probes) for 2 h at RT, and stained with Hoechst 33342 (Thermo Fisher Scientific, Waltham, MA, USA).

### Image acquisition and analysis

All images were captured with a confocal microscope, LSM 710 (Zeiss, Jena, Thüringen, Germany). To analyze the tangential migration of E13.5 mouse embryos, VGAT^Venus^ and Hoechst images were captured with a 20×/0.8 numerical aperture (NA) objective. To quantify tangential migration of TS2‐neo mice in the neocortex on E13.5, tangentially aligned five equidistance bins were set from the corticostriatal boundary to the cortical arch, and Venus‐positive migrating cells were counted in each equidistance bin using imagej (NIH, Bethesda, MD, USA) (Fig. 4C,D). Similarly, for the quantification of neuronal distribution, a defined region at the entrance of the neocortex was separated by radially aligned six equidistance bins and migrating cells were counted in each equidistance bin (Fig. 4E). For presynaptic puncta analysis in the primary somatosensory cortex at P21, L2 and L4 were captured with a 63×/1.4 NA objective. Acquired images were deconvolved with imagej plugin software, iterative deconvolve 3d [25]. The number of presynaptic puncta was automatically counted, and puncta density and area were measured in each image.

### Statistical analyses

Unpaired two‐tailed *t*‐tests with Welch's correction, paired two‐tailed *t*‐tests, and Kolmogorov–Smirnov (K‐S) tests were performed with graphpad prism 7.0 (GraphPad Software, San Diego, CA, USA). The data were expressed as mean ± standard error.

## Results

### TS2‐neo mice show normal learning flexibility and high social competitive dominance

Previous studies reported that the TS2‐neo mice performed normally in spatial learning and memory, and showed repetitive/restricted behavior and impaired social interaction, dominant in a tube test [4, 5]. To address behavioral phenotypes in a social domain, the feature of the IntelliCage system was utilized, that is, a group‐housed condition. TS2‐neo mice were simultaneously tested with WT mice in the same IntelliCage (Fig. 1A).

Following acclimation, the spatial leaning‐based behavioral sequencing task consisting of an acquisition phase and three reversal phases was performed (Fig. 1B,C). In this study, the TS2‐neo and WT mice equally acquired the spatial leaning‐based behavioral sequencing task (the acquisition phase) (Fig. 1D,E). Further, TS2‐neo mice showed no obvious abnormality during reversal learning blocks, indicating normal behavioral flexibility in a setup that did not require any handling (Fig. 1D,E).

**Fig. 2:**
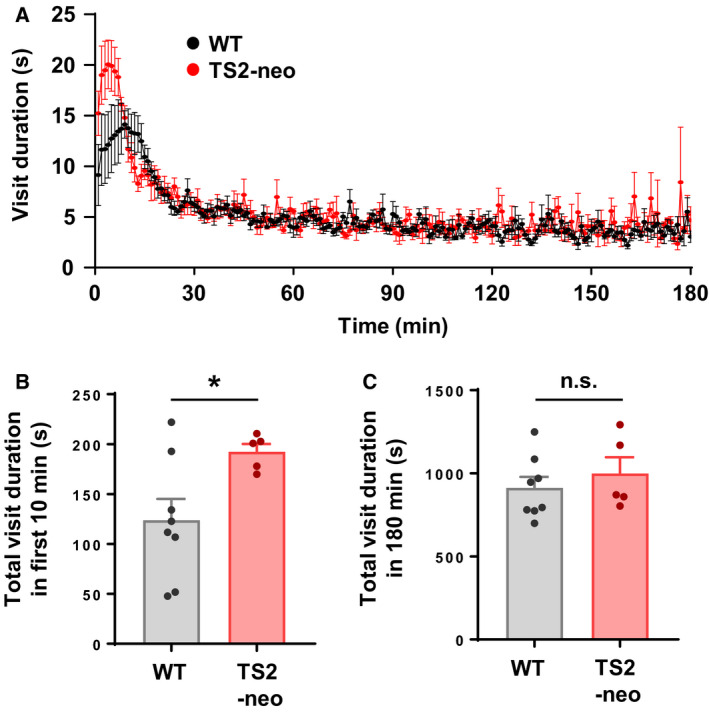
TS2‐neo mice showed highly competitive dominance in the IntelliCage. (A) Average visit duration per minute at the active rewarded corners throughout each 3‐h session from day 1 to day 20 during the acquisition block. (B) Averaged visit duration at the water available corner in the first 10 min after the start of water availability from day 16 to day 20 during acquisition. (C) Average total visit duration throughout the 3‐h session from day 16 to day 20 during acquisition. *n* = 8 mice in WT and *n* = 5 in TS2‐neo mice. Data are mean ± SEM, *P < 0.05, n.s., not significant; unpaired two‐tailed *t*‐test with Welch's correction (B, C).

In social domain of mice, social competitive dominance behavior was assessed in this study. At the start of each session of the behavioral test, the TS2‐neo and WT mice were observed to make intensive corner visits competing against each other to get water as a reward throughout the test. We also found that the duration of corner visits peaked earlier in TS2‐neo than in WT mice (Fig. 2A). TS2‐neo mice displayed significantly longer durations in the first 10 min of the last five sessions than did WT mice (Fig. 2B; *t*(11) = 3.005, df = 8.71, *P* < 0.05). The duration of corner visits through session (3‐h) was not different between groups (Fig. 2C; *t*(11) = 0.7375, df = 7.611, *P* = 0.48), indicating that motivation for water rewards was not affected in TS2‐neo mice. These findings demonstrated that TS2‐neo mice showed high social competitive dominance status in a group‐housed condition.

### Excess inhibitory synapses in TS2‐neo mouse cortex

**Fig. 3:**
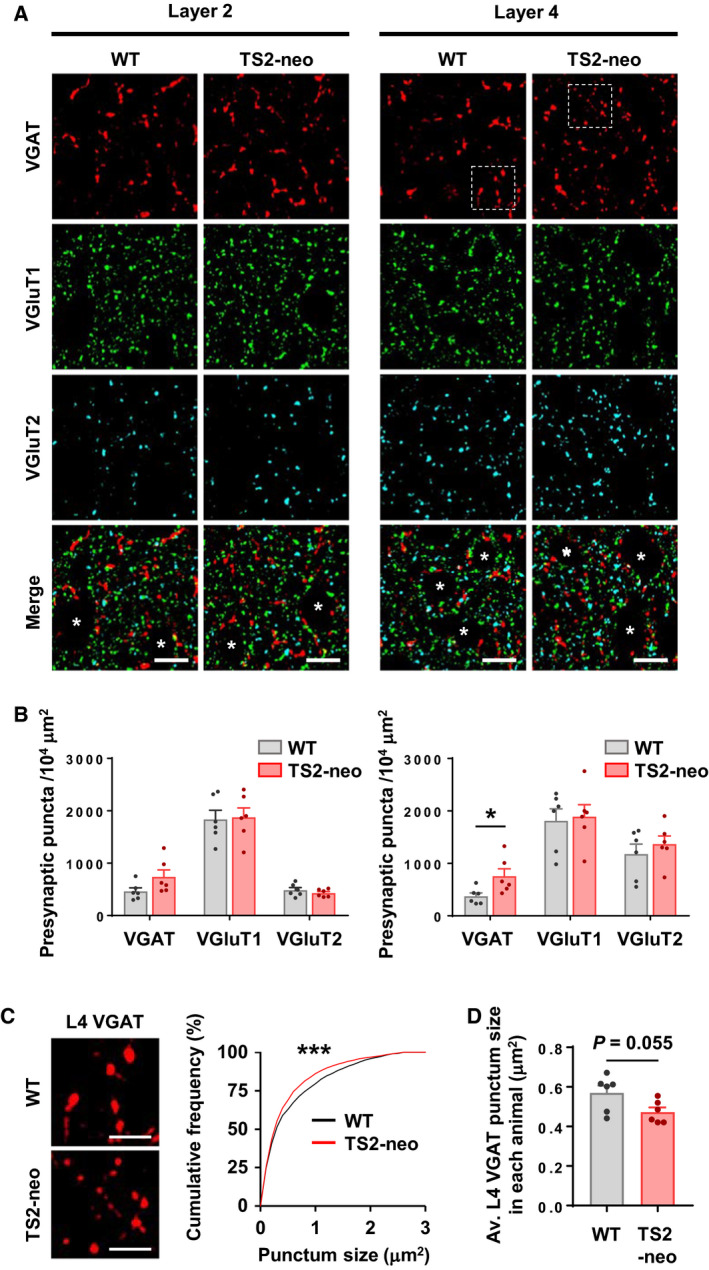
Excess inhibitory synapses in TS2‐neo mice during neural circuit development. (A) Deconvoluted images of presynaptic markers in the primary somatosensory cortex at P21. Asterisks indicate cell bodies. (B) VGAT puncta density was increased at L4 of TS2‐neo mice (right), but not at L2 (left). (C) Enlarged images of VGAT puncta corresponding to the dotted squares in A (left). Size of L4 VGAT puncta was decreased in TS2‐neo mice (right). (D) Average size of L4 VGAT puncta in each animal. *n* = 6 mice per group. Data are mean ± SEM, *P < 0.05, ***P < 0.001; unpaired two‐tailed *t*‐test with Welch's correction (B, D) and Kolmogorov–Smirnov test (C). Scale bars, 10 μm (A), 5 μm (C).

After investigating the behavioral phenotype of TS2‐neo mouse, the alteration of inhibitory neuronal circuit formation was explored. A tight E/I balance in synaptic transmission and neural circuits are a key component of brain development and function, and growing evidence implicates disturbed E/I balance in the pathophysiology of ASD. To understand impacts on inhibitory synapses, presynaptic puncta labeled with presynaptic markers—vesicular GABA transporter (VGAT) and vesicular glutamate transporter (VGluT) 1 and 2 in the barrel somatosensory cortex at P21, were assessed. By P21, the critical period for developmental synaptic formation and elimination is completed in the barrel field [26]. VGAT‐expressing inhibitory synaptic terminals and VGluT1/2 [27]‐expressing excitatory synaptic terminals were counted (Fig. 3A,B). No obvious difference was observed in L2, but VGAT‐positive synaptic density was increased in L4 of TS2‐neo mice (Fig. 3B, right; *t*(10) = 2.497, df = 6.905, *P* < 0.05). In contrast, no differences in VGluT1/2‐positive presynaptic puncta in either L2 or L4 were noted. The measurement of punctum size revealed the existence of smaller presynaptic VGAT puncta in L4 of the somatosensory cortex in TS2‐neo mice (Fig. 3C; K‐S: *D* = 0.8387, *P* < 0.0001, Fig. 3D; *t*(10) = 2.227, df = 8.384, *P* = 0.0551), indicating functional differences in inhibitory neurotransmission induced by aberrant presynaptic inputs. These histological results suggest the existence of altered synaptic E/I balance in the neocortex of young adolescent TS2‐neo mice, which may underlie autistic phenotypes observed in this mouse model.

### Altered distribution of immature inhibitory neurons during neural circuit formation

TS with a G406R mutation in exon 8A or 8 is classified as TS type 1 or 2, respectively. GABAergic interneurons derived from pluripotent stem cells of TS type 1 patients show delayed neuronal migration [19]. Impacts of TS type 2 mutation on inhibitory neurons *in vivo* were assessed in TS2‐neo mice to address whether numbers of inhibitory neurons during embryonic neuronal migration are affected.

**Fig. 4:**
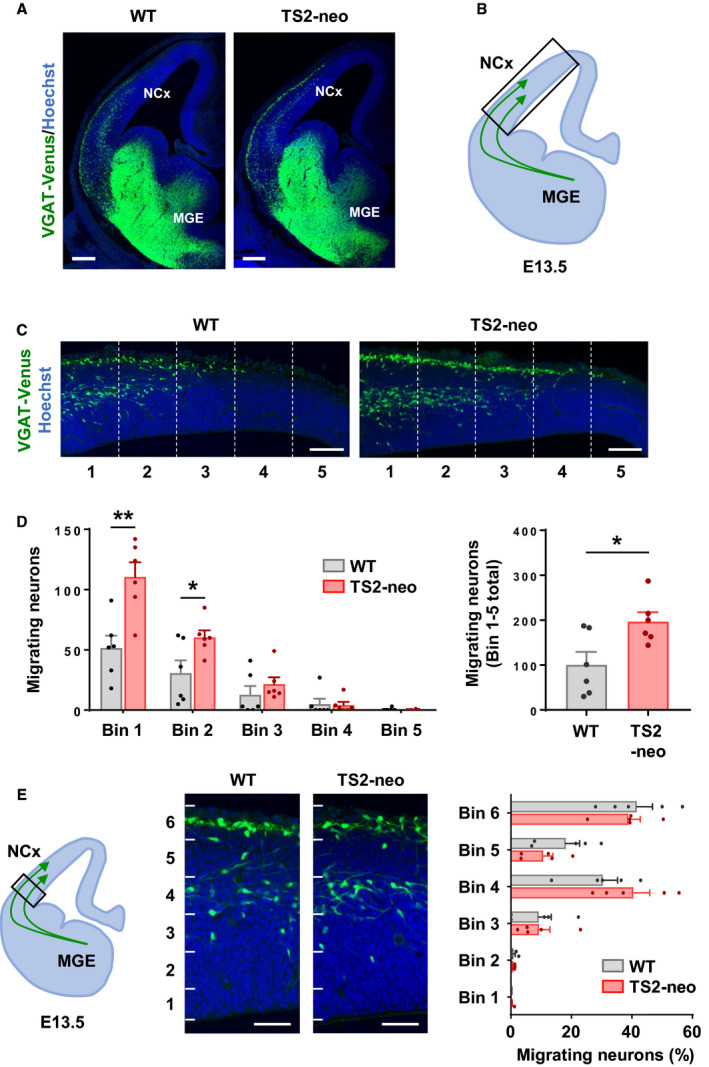
Excess migrating inhibitory neurons in TS2‐neo embryonic brain. (A) Coronal sections of the telencephalon at E13.5. Immature inhibitory neurons were labeled with Venus. (B) Schematic illustration of tangential migratory streams derived from the medial ganglionic eminence (MGE) to the neocortex (NCx). The box shows the region of interest presented and analyzed in C and D. (C) Representative images of migrating neurons in the embryonic neocortex. (D) Equidistance bin analysis revealed an increase in migrating neurons labeled with Venus that reached the neocortex in TS2‐neo mice (*n* = 6 embryos per group). (E) Number of Venus‐positive neurons in radially aligned six equidistance bins (*n* = 6 embryos per group). Data are mean ± SEM, *P < 0.05, **P < 0.01; unpaired two‐tailed *t*‐test with Welch's correction (B, C). Scale bars, 200 μm (A), 100 μm (C, E).

To analyze neuronal development in embryos, immature inhibitory neurons were visualized by crossing TS2‐neo with VGAT^Venus^ mice. We did not observe gross abnormality in the brain size as well as the distribution of Venus‐positive cells in the medial ganglionic eminence in TS2‐neo mice compared to WT at E13.5 (Fig. 4A). However, we found that excess migrating inhibitory neurons reached the neocortex in TS2‐neo mouse embryos (Fig. 4B–D; Bin 1: *t*(10) = 3.717, df = 9.72, *P* < 0.01; Bin 2: *t*(10) = 2.456, df = 7.814, *P* < 0.05; Bin 1–5 total: *t*(10) = 2.717, df = 9.154, *P* < 0.05), while migratory routes were not affected (Fig. 4E). Body weight (WT: 0.726 ± 0.016 g, *n* = 16, TS2‐neo: 0.757 ± 0.016 g, *n* = 8, *P* = 0.19) and brain weight (WT: 0.055 ± 0.001 g, *n* = 16, TS2‐neo: 0.055 ± 0.001 g, *n* = 8, *P* = 0.80) were not significantly changed in TS2‐neo mouse embryos at E17.5, showing a lack of gross developmental deficits in TS2‐neo mice. These observations suggest that tangential migration and/or complimentary developmental steps, such as proliferation and differentiation, were altered in TS2‐neo mouse embryonic brain, which may subsequently affect the maturation of inhibitory circuit later during the prenatal and postnatal period.

## Discussion

In this study, a behavioral evaluation of TS2‐neo mice revealed high social competitive dominance status in a group‐housed condition, without obvious deficits in the acquisition of behavioral sequencing. In addition, the development of inhibitory neuron was investigated, which has not been studied so far in TS2‐neo mice. We found that TS2‐neo mice showed histological abnormalities in inhibitory neurons during development, increased numbers and decreased size of inhibitory presynaptic terminals in young adolescent animals, and a higher number of inhibitory neurons migrating from the medial ganglionic eminence to the neocortex at E13.5.

The IntelliCage has been widely used to assess chemical, environmental, and genetic mouse models for neuropsychiatric diseases [20, 21, 28, 29, 30]. This system allows observation of mouse behavior while minimizing human intervention and isolation stress. Social–behavioral abnormality was identified in TS2‐neo mice, namely social competitive dominance in this study. In regard to social domain of mouse behavior, altered social behavior in TS2‐neo mice is reported in conventional behavioral tasks relevant to ASD [4, 5]. TS2‐neo mice are also reported to win significantly more trials on a tube test, to find winner and looser at one‐on‐one duel [5], and the present results can be said to be a reconfirmation data of the study. In addition, competitive dominance behaviors in the IntelliCage are observed not in a duel but in a group‐housed condition: Multiple opponents were the cagemates, there were four options (four corners), and low‐dominance mice could get rewards after several minutes. Thus, high‐dominance status can be concluded as the phenotype of TS2‐neo mice.

The study of social dominance in ASD patients is still limited, and a few studies have suggested an atypical way of recognition in these patients when they were asked to judge who is dominant between two persons presented as schematic diagrams or animations [31, 32]. Further, a series of studies reported dominant social phenotypes in multiple rodent models associated with ASD [33, 34, 35]. Thus, current observations support and extend autistic traits of TS2‐neo mice using the IntelliCage apparatus.

The dysfunction of inhibitory neural circuits is a common feature in ASD [13, 14]. Synaptic physiological studies on genetic ASD mouse models reported mixed results for E/I imbalance. Many studies reported reduced inhibition; however, others suggested decreased excitation or increased inhibition [13, 14]. In the present study, differences were found in the density and the size of inhibitory presynaptic puncta labeled by VGAT in L2 of the somatosensory cortex at P21. Although L‐type Ca^2+^ channels may regulate both excitatory and inhibitory synapse maturation and mediate activity‐dependent synaptic modulation [11, 12], differences were not observed in excitatory synapses in this study. Interestingly, specific changes to inhibitory synapses were reported in another knock‐in mouse model, where an ASD‐linked neuroligin‐3 R451C mutation increases inhibitory synaptic transmission with no apparent effect on excitatory synapses [36]. This result is reported despite the finding that neuroligin‐3 is expressed in both inhibitory and excitatory neurons. In TS2‐neo mice, the density of VGAT puncta increases, but the size of puncta becomes smaller, assuming more frequent but weaker inhibitory synaptic transmissions. Further electrophysiological studies are needed to demonstrate the synaptic functional significance of these anatomical observations.

In addition to postnatal synaptic maturation, the activity of L‐type Ca^2+^ channels can regulate inhibitory neuron migration during development [10, 19]. Abnormality in such migration is also associated with ASD [37]. Facilitation of inhibitory neuron migration in TS2‐neo mice observed in this study contrasts with delayed migration phenotype of inhibitory neurons derived from pluripotent stem cells of TS type 1 subjects [19]. These cells bear the G406R mutation in the exon 8A encoding Ca_v_1.2, the alternative exon of exon 8 where the mutation is introduced into the TS2‐neo mouse. To explain inconsistent phenotypes of TS mutations, consideration of expression changes of Ca_v_1.2 splicing isoforms is needed. The expression of the exon 8A containing isoform is initially higher during midembryonic development until about E14 in the mouse brain. Then, the expression of the exon 8 containing isoform increases especially after birth, resulting in a much more dominant expression of this isoform in mature neurons both in humans and in mice [1, 17, 38]. Also, in TS2‐neo mice, an inversed neomycin cassette kept in exon 8A reduced the expression of both isoforms expressed from the heterozygous knock‐in allele [4]. Consistent with this original report, we confirmed lowered expression of exon 8 with G406R mutation in TS2‐neo mice (data not shown). These physiological and experimental differences in mutant channel expression may influence the migration of inhibitory neurons and produce distinctive results between experiments.

Together with several developmental abnormalities reported in excitatory neurons, the results of this study suggest that the alteration of the development of inhibitory neurons induces E/I imbalance in TS2‐neo mice that may explain a circuit abnormality underlying ASD‐associated behavior. One possible molecular mechanism downstream of altered intracellular Ca^2+^ dynamics triggered by G406R mutation in L‐type Ca^2+^ channel can be explained by the dysregulated activation of multifunctional Ca^2+^/calmodulin‐dependent protein kinases (CaMKs), that is, CaMKI, II, and IV, whose activities are tightly regulated by neuronal Ca^2+^. CaMKs have various downstream substrates and play key roles in neural circuit development, synaptic plasticity, and cognition, as well as in the pathophysiology of neuropsychiatric disorders [39]. Subsequent studies addressing when, where, and how the G406R mutation acts to affect neuronal circuit development and the resulting ASD‐associated behaviors of the mice will be needed to understand the complex pathophysiology of TS symptoms.

## Conflict of interest

The authors declare no conflict of interest.

## Author contributions

SH, MK, and ST‐K supervised the projects and wrote the manuscript, with input from all authors. YO, JZ, PM, SU, SN, and YY contributed to histological analysis. HT, AH, and MK performed and analyzed behavioral phenotyping. All authors read and approved the final manuscript.
